# Environmental sustainability of post-orthodontic dental retainers: a comparative life-cycle assessment of Hawley and Essix retainers

**DOI:** 10.1093/ejo/cjae012

**Published:** 2024-03-15

**Authors:** Timothy You Da Tan, Brett Duane, Ahmed Hussein, Anna Samsonova, Gwenola Sizun, Loujin Shakerdi, Roqaya Taqi, Samuel Wolfram, Nazanin Ziaeefard, Darius Sagheri

**Affiliations:** School of Dental Science, Trinity College Dublin, Dublin, Ireland; School of Dental Science, Trinity College Dublin, Dublin, Ireland; School of Dental Science, Trinity College Dublin, Dublin, Ireland; School of Dental Science, Trinity College Dublin, Dublin, Ireland; School of Dental Science, Trinity College Dublin, Dublin, Ireland; School of Dental Science, Trinity College Dublin, Dublin, Ireland; School of Dental Science, Trinity College Dublin, Dublin, Ireland; School of Dental Science, Trinity College Dublin, Dublin, Ireland; School of Dental Science, Trinity College Dublin, Dublin, Ireland; School of Dental Science, Trinity College Dublin, Dublin, Ireland

**Keywords:** sustainability, orthodontic retainers, life-cycle assessment, carbon footprint, environment

## Abstract

**Background:**

Environmental sustainability has been brought into the limelight due to the global climate crisis. This crisis is driven by human activities and even the healthcare sector is no exception. Within dentistry, orthodontics is a large global market; hence, the use of post-orthodontic retainers has a significant environmental footprint. The aim of this study was to determine the environmental sustainability of post-orthodontic retention using Hawley and Essix retainers.

**Materials and methods:**

A comparative life-cycle assessment (LCA) was carried out to compare the environmental impact of both retainers. All inputs and outputs were accounted for using the Ecoinvent database, v3.7.1, and openLCA software. Sixteen impact categories were used to determine their environmental burden.

**Results:**

Of the 16 impact categories, the Hawley had a greater environmental burden than the Essix retainer in 12 categories. The Hawley’s most significant contributors to its impact values are factory manufacturing and in-house production, with an average of 41.45% and 52.52%, respectively. For the Essix, the greatest contributor is factory manufacturing, with an average of 64.63%. However, when factoring in the lifespan of the retainers, the Essix would have a greater environmental impact than the Hawley retainer.

**Limitations:**

This study employed a comparative LCA. There were also assumptions made, but these were supported by research.

**Conclusions:**

On the basis of the evidence gathered in this study, Hawley retainers are more environmentally sustainable than Essix retainers. These results would better enable clinicians to factor in the environmental impact and make informed decisions on the choice of retainer type.

## Introduction

Environmental sustainability is one of the most pressing issues of our time. Climate change is increasing at unprecedented levels and has left observable consequences [1–4]. In response, numerous global plans, initiatives, and goals have been implemented [5, 6].

The healthcare sector is estimated to contribute to about 4.4% of global net emissions, equivalent to 2 gigatons of carbon dioxide [7, 8]. In Ireland, the healthcare footprint stands at 4.4% of the national footprint [7]. The bulk of healthcare emissions are attributed to the supply chain and the use of energy-guzzling equipment [7]. To address this, the Health Service Executive (HSE) is developing a climate change plan [9], beginning with decarbonizing HSE infrastructures [10, 11]. Although there have been several plans and efforts to mitigate climate change, we still have a long way to go before any significant impact can be achieved [12].

In dentistry, one way to increase environmental sustainability is through sustainable procurement of products [9]. To achieve the ideal straight teeth for that perfect smile, more people are receiving orthodontic treatment [13, 14]. Furthermore, orthodontic treatment modalities have evolved over the years, allowing people to opt for alternatives like clear aligners [15]. In 2021, the global orthodontic market stands at USD 5.38 billion and is expected to increase by 17.2% between 2021 and 2028 [14]. Long-term post-orthodontic retention is an integral part of orthodontic treatment, preventing any relapse of the final occlusal outcome [16]. With the rise in demand for orthodontic treatment, it also means the rise in the need for post-orthodontic retainers. Lifelong retention entails that lost or worn retainers need to be replaced. The constant need to replace retainers for long-term retention results in cumulative environmental ramifications that need to be understood for clinicians to make informed decisions when selecting the most appropriate retainer for their patients.

Environmental impact can be measured in several different ways like the more basic carbon foot printing or using a more comprehensive life-cycle assessment (LCA). An LCA is more advantageous as it provides disability-adjusted life years (DALYs) and a total of 16 impact categories. LCA, commonly referred to as a ‘cradle to grave’ analysis, is a technique used to evaluate the cumulative environmental impact of a product through its life cycle. These include any activity from gathering of raw materials, manufacturing, transportation, consumer use, and means of disposal [17]. LCA comprehensive evaluation quantifies the emissions data into different impact categories like climate change, ozone depletion, acidification, and eutrophication. This makes the data from each product comparable and easier to analyse. This gives the researcher a more holistic view of the environmental impact [18, 19].

The aim of this study was to determine the environmental sustainability of post-orthodontic retention of two widely used dental retainers, namely Hawley and Essix retainers. A comparative LCA was conducted on both types of retainers with common steps and procedures excluded. Overall, it is hoped that this information will allow clinicians to better understand the environmental impact of dental retainers and subsequently make informed decisions in terms of the choice of retainers.

## Materials and methods

### Functional unit

The functional unit of this study was the fabrication of one orthodontic retainer for post-orthodontic retention. The following types of retainers were compared: (1) Hawley retainer and (2) Essix retainer. The products chosen were based on those currently in use at Dublin Dental University Hospital (DDUH) and the brands were anonymized.

### System boundaries

**Figure 1.**
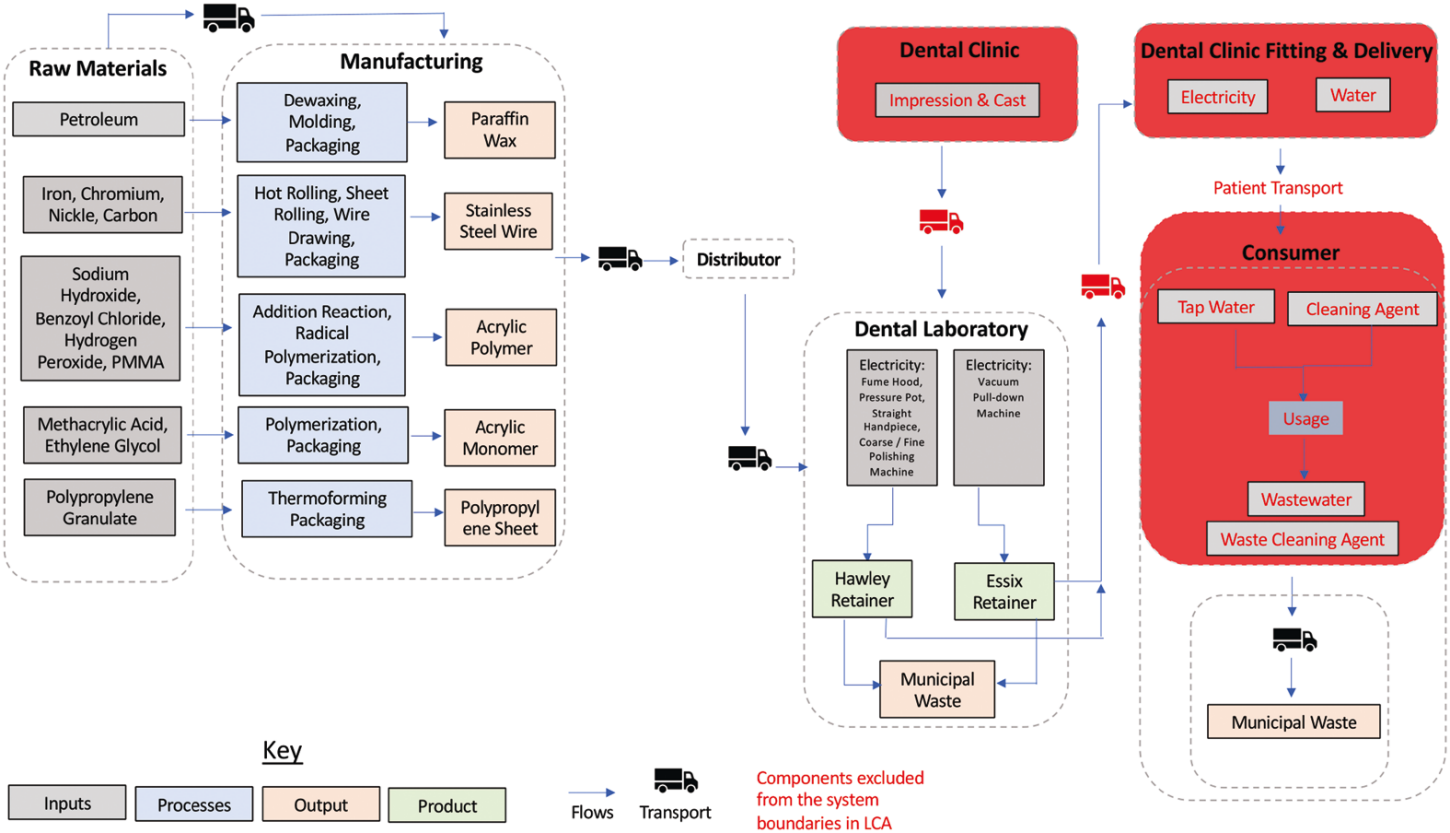
System Boundaries.

The entire product system was considered, including the geographic location of the manufacturer (Fig. 1). Impressions and cast made at the initial dental visit, fitting and delivery of appliance as well as consumer use were excluded. Information on raw materials and manufacture was obtained online based on information from the distributors of the orthodontic components, which were gathered from the dental production laboratory in DDUH. Items that could not be sourced back to the manufacturer were replaced by suitable substitutes based on estimates of similar products. Estimates of power usage during the final product manufacture were obtained from machinery specifications taken from the machines used in DDUH.

### Assumptions and exclusions

A detailed list of assumptions and exclusions made in this study is summarized in Supplementary Table 1.

#### Raw materials

The Essix retainer is made from 1-mm polypropylene sheets that are thermoformed while the Hawley retainer is made of several components that require more processes to produce the final product. These components are stainless-steel wires to form Adam’s cribs and labial bow, liquid and powdered acrylic to make the base, and modelling wax to hold the stainless-steel wires in place during the manufacturing process (Tables 1 and 2). Raw materials used for common steps of both Essix and Hawley retainers were excluded from this comparative LCA (Table 3).

**Table 1.** Products specific to Essix retainer in fabrication.

Product	Product name	Quantity	Unit
Acrylic sheet	Essix Ace Plastic (Pctg) 1 mm	20.26	g

**Table 2.** Products specific to Hawley retainer in fabrication

Product	Product name	Quantity	Unit
Stainless-steel wire	Stainless steel spring hard wire 0.7mm	0.36	g
Wax	Antuex modelling wax	0.60	g
Powdered acrylic	MP2 orthodontic cold cure acrylic powder 9 kg	4.47	g
Liquid acrylic	MP2 orthodontic cold cure acrylic clear liquid 5 L	1.76	ml

**Table 3.** Common products used for fabrication of both Essix and Hawley retainers

Generic product	Product name	Quantity	Unit
Impression material	Alginate Aroma fast set fine	25.99	g
Impression tray	Transform Tray Dentate	1	
Tray adhesive	DEHP Tray Adhesive Liquid	1	ml
Disinfection	Euro Sept Plus Impression Disinfection	1L changed daily, used 100’s of times	l
Type 3 dental stone	Elite Arti Articulating Stone	44.00	G
Type 1 dental stone	SLX Plaster Type 1	8.60	G
Water	-	153.7	ml
Scalpel	Swann-Morton, disposable scalpels	1	
Polishing burs	Dialite Polishing Kit	1	
Pumice	Quartz-free pumice powder		

Weights of each individual raw material used to make an Essix and a Hawley retainer and packaging material were measured to the nearest 0.01 g using an electronic weight scale (OHAUS Scout™ SKX Portable Balance SKX2202).

#### Manufacture and packaging

All manufacturing processes of the initial raw materials by the manufacturing companies prior to DDUH dental laboratory were accounted for in the comparative LCA (Supplementary Tables 2 and 3).

The final production of each type of retainer was based on shadowing a third-year dental technician student from the DDUH through each step of the production process. The data were recorded accordingly.

The final production was estimated using kWh based on the machinery used in DDUH (Table 4). It was assumed that 0.00754 and 0.190122 kWh were needed to produce an Essix and a Hawley in the DDUH dental laboratory, respectively. For both types of retainers, energy from machineries used for common steps in production was excluded (Table 4). The maintenance of the machines and waste generated by the machines were excluded. Excess materials that were trimmed off during the production process like acrylic sheets, stainless steel, and modelling wax were assumed to be disposed of as municipal waste. Powdered and liquid acrylic were assumed to be used in precise amounts, with little waste. The acrylic waste was assumed to be sent to municipal waste but the environmentally harmful acrylic liquid fumes (0.00081 g) generated were accounted as methyl methacrylate emissions.

**Table 4.**
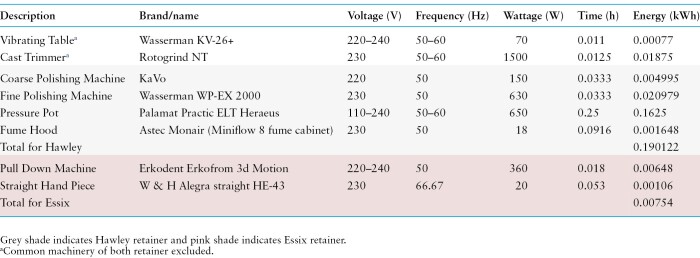
Energy consumed in the manufacturing of Essix and Hawley in laboratory fabrication

The packaging used in transport of raw materials to the manufacturers was excluded. The packaging for transport of products from the manufacturers to distributors was assumed to be similar to the packaging used to transport products from the distributors to DDUH. Hence, the disposal of the packaging was only accounted for once. All products were assumed to be transported in a 450-g corrugated cardboard box. The number of products in each box was assumed based on volumetric calculations. All instruction manuals in the packaging were excluded.

#### Transport

The supply chain between the factory and DDUH was assumed to go via a distributor. All transport routes were assumed to be via the shortest land, sea, and air routes, measured in kg km. Distances were measured using Google Maps®, Air Miles®, and Sea Rates®.

For the Essix retainer, its main component is the acrylic sheets. The acrylic sheets were assumed to be transported from the factory in Northwich, UK, to the distributor in Uxbridge, UK, via land using an EURO6 engine lorry. From the distributor, the sheets were transported via an EURO6 lorry to Holyhead port and then by a container ship to Dublin port. The materials were then transported via an EURO6 lorry from Dublin port to the distributor’s Ireland headquarters in Meath. From here, the sheets were distributed to DDUH via a light commercial lorry.

For the Hawley retainer, there are several components involved, namely stainless-steel wires, modelling wax, powdered acrylic, and liquid acrylic. Stainless-steel wires were assumed to be transported from the factory in Madrid, Spain, to the distributor in West Yorkshire, UK, via land using an EURO6 lorry. Powdered and liquid acrylic were transported from the factory in Dorset, UK, and transported via land using an EURO6 lorry to the same distributor in West Yorkshire, UK. From the distributor, these products were transported via an EURO6 lorry to Holyhead port and then by a container ship to Dublin port. The materials were then transported via an EURO6 lorry from Dublin port to the distributor’s Ireland headquarters in Bray. From here, the materials were distributed to DDUH via a light commercial lorry. For Beauty wax, it was transported from the factory in Dorset, UK, to the distributor in Swindon, UK. From the distributor, the beauty wax was transported via an EURO6 lorry to Holyhead port and then by a container ship to Dublin port. The materials were then transported via an EURO6 lorry from Dublin port to the distributor’s Ireland headquarters in Kinsale, Cork. Thereafter, the materials were distributed to DDUH via a light commercial lorry.

#### Consumer use and disposal

In terms of consumer use, water and cleaning agents used by the consumer for daily maintenance of the retainers were excluded from the comparative LCA, as both retainers require it. The retainers were assumed to be disposed of in domestic waste upon usage until failure by the patient.

#### Data collection and analysis

A life-cycle inventory was produced using the assumptions above and a breakdown of the two products can be seen in Supplementary Tables 2 and 3. The reference database Ecoinvent, v3.7.1, was used alongside openLCA, v1.10.3, for the comparative life-cycle analysis according to International Organisation for Standardisation (ISO) and Product Environmental Footprint (PEF) standards [20, 21]. The life-cycle impact assessment (LCIA) methods and impact categories were based on PEF standards, as described in Table 5. A contribution analysis for each retainer was conducted. Disability-adjusted life years (DALYs) were produced using a ReCiPe H Endpoint and converted into seconds.

**Table 5.** Impact categories and life-cycle impact assessment methods used in this study.

Impact category	LCIA method (units)	Description
Acidification	ILCD 2011 Midpoint + (mol H + eq)	Acidification of soils and freshwater due to gas release
Climate change	IPCC 2013 GWP 100a (kg CO_2_-eq)	Potential for global warming from greenhouse gas emissions
Ecotoxicity, freshwater	ILCD 2011 Midpoint + (CTUe)	Harmful effects of toxic substances on freshwater organisms
Eutrophication, freshwater	ILCD 2011 Midpointþ (kg P-eq)	Changes in freshwater organisms and ecosystems caused by excess nutrients
Eutrophication, marine	ILCD 2011 Midpoint + (kg N-eq)	Changes in marine organisms and ecosystems caused by excess nutrients
Eutrophication, terrestrial	ILCD 2011 Midpoint + (mol N eq)	Changes in land organisms from excess nutrients in soil and air
Human toxicity, cancer	ILCD 2011 Midpoint + (CTUh)	Harm to human health that causes or increases cancer risk
Human toxicity, non-cancer	ILCD 2011 Midpoint + (CTUh)	Harm to human health that is not related to cancer or ionizing radiation
Ionising radiation	ILCD 2011 Midpoint + (kBq U^-235^ eq)	Potential damage to human DNA from ionizing radiation
Land use	Soil quality index based on LANCA (points)	Depletion of natural resources, change in soil quality and reduction in biodiversity
Ozone depletion	ILCD 2011 Midpoint + (kg CFC11 eq)	Air emissions causing stratospheric ozone layer destruction
Particulate matter	PM method (disease incidence)	Harm to human health caused by particulate matter emissions (respiratory inorganics)
Photochemical ozone formation	ILCD 2011 Midpoint þ (kg NMVOC-eq)	Harm to human health from gas emissions that contribute to smog in the lower atmosphere
Resource use, fossils	CML-IA baseline (MJ)	Depletion of natural fossil fuels
Resource use, minerals and metals	CML-IA baseline (kg Sb-eq)	Depletion of natural non-fossil fuel resources
Water use	AWARE (m^3^ deprivation)	Potential for water deprivation to humans and ecosystems globally

## Results

### Life-cycle impact assessment

The comparative LCIA results for each type of retainer are shown in Table 6. In the 16 impact categories, the Hawley retainer had a greater environmental burden than the Essix retainer in 12 categories. In these 12 categories, the difference ranged from a 104% increase (fossils) to a 229% increase (carcinogenic effects).

**Table 6.** Life-cycle impact assessment results; summary of environmental burdens for the use of an Essix or a Hawley retainer.

*Impact Category*	*Units*	*Essix*	*Hawley*
Non-carcinogenic effects	CTUh	6.93E-09	9.69E-09
Minerals and metals	kg Sb-eq	4.22E-07	7.79E-07
Freshwater ecotoxicity	CTU	0.11191	0.08261
Climate change total	kg CO_2_-eq	0.10914	0.15337
Freshwater eutrophication	kg P-eq	2.34E-05	2.25E-05
Respiratory effects—inorganic	Disease incidence	3.32E-09	3.61E-09
Photochemical ozone creation	kg NMVOC—eq	0.00028	0.00037
Terrestrial eutrophication	mol N-eq	0.00081	0.00105
Marine eutrophication	kg N-eq	8.89E-05	0.00011
Ozone layer depletion	kg CFC-11-eq	3.13E-09	6.51E-09
Ionizing radiation	kg U235-eq	0.00585	0.00736
Land use	Points	1.07179	0.83699
Freshwater terrestrial acidification	mol H+-eq	0.00038	0.00066
Fossils	MJ	2.34237	2.44299
Carcinogenic effects	CTUh	1.57E-09	3.60E-09
Dissipated water	m^3^ water-eq	0.06575	0.0191

The Hawley retainer released 0.15337 kg of CO_2_ per retainer made, approximately 1.4 times more than the Essix retainer. The amount of trichlorofluoromethane produced by the Hawley retainer resulting in ozone layer depletion was approximately 2.1 times more than the Essix retainer.

### Contribution analysis

**Figure 2.**
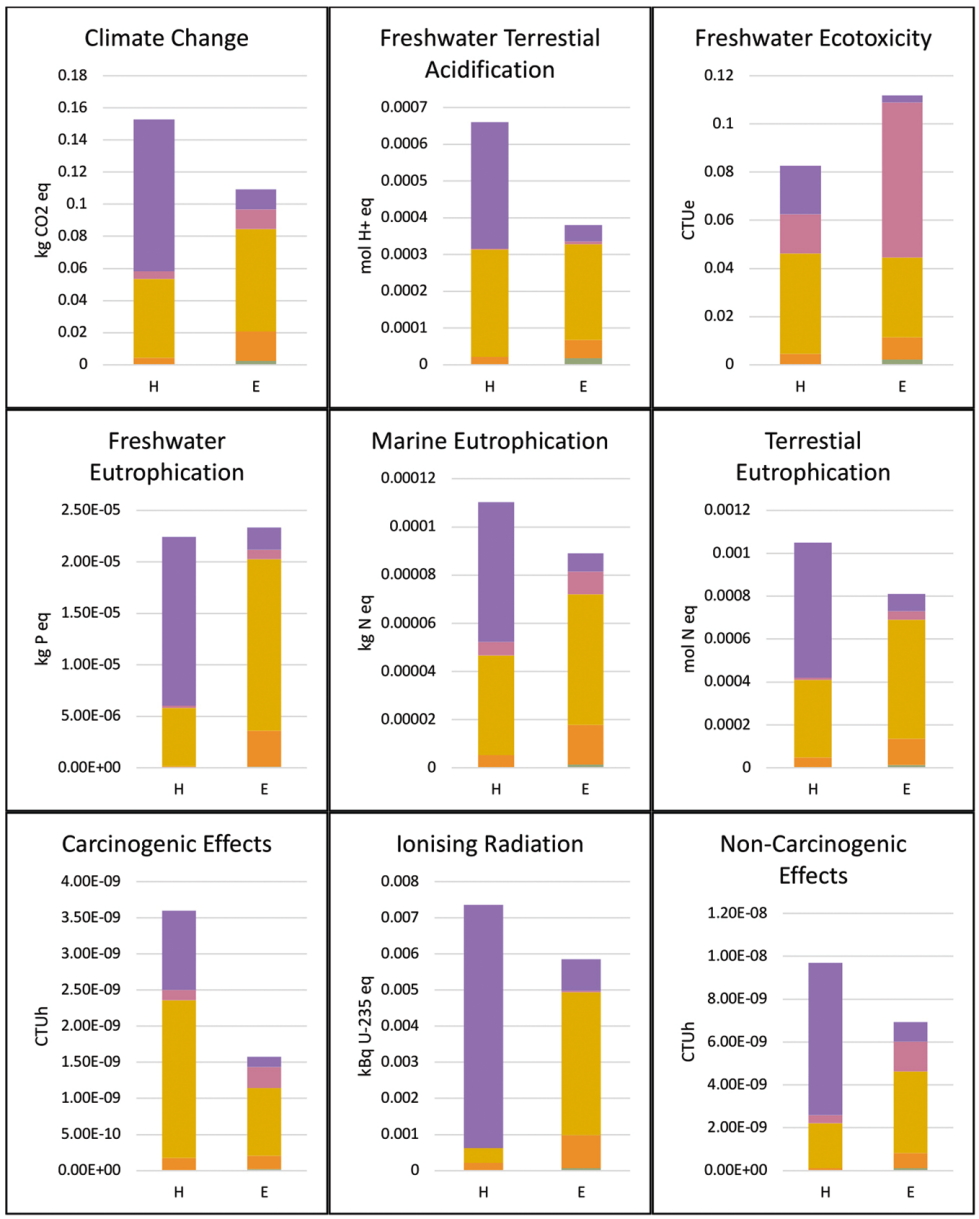

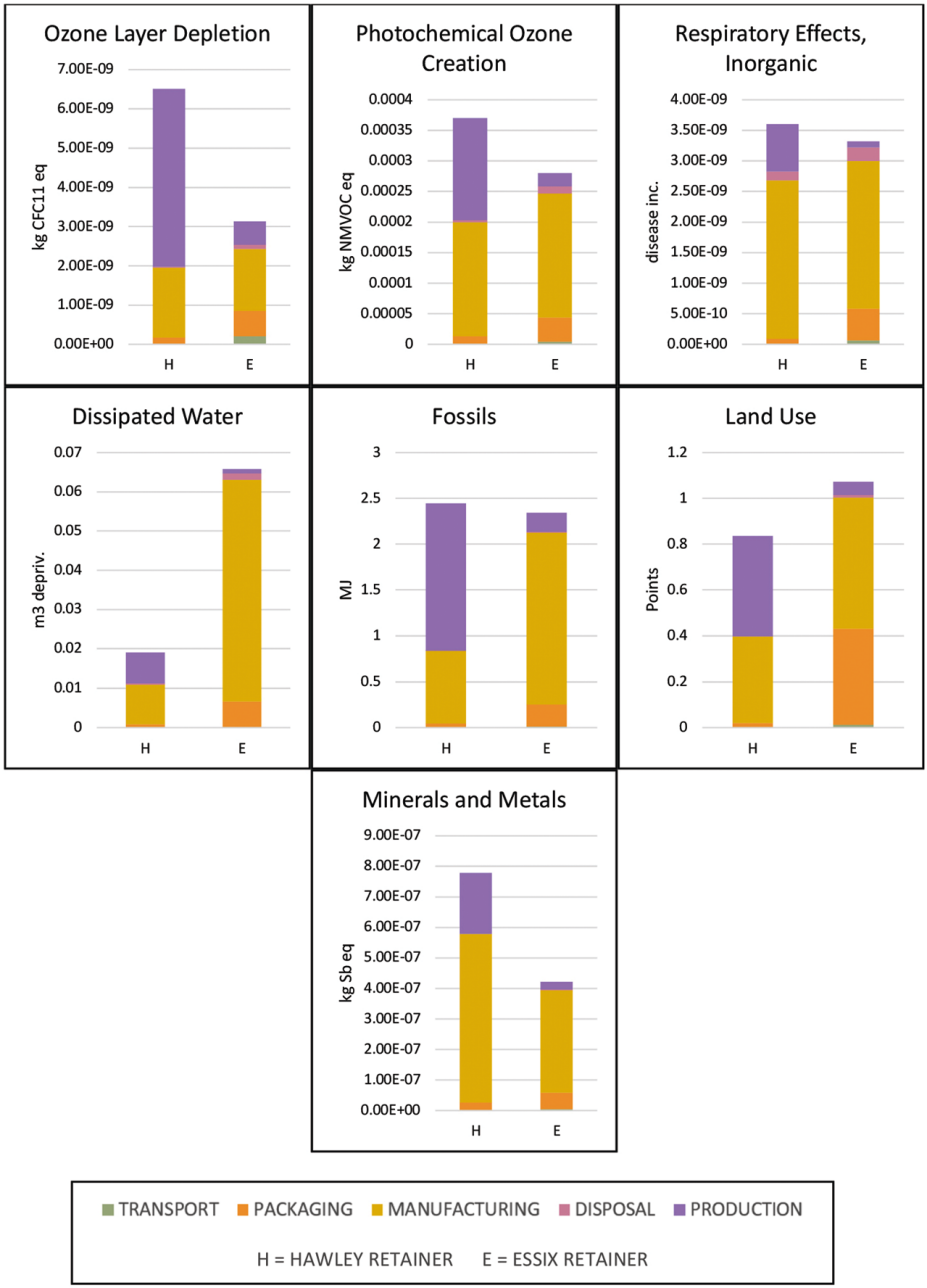
LCIA Results.

The contribution analysis highlights how each aspect of the life cycle contributed to the final comparative LCIA results and is shown in Fig. 2.

The Hawley’s most significant contributors to its impact values are factory manufacturing and in-house production in the DDUH dental laboratory, giving average contributions of 41.45% and 52.52%, respectively. For the Essix, the main contributors were factory manufacturing and packaging, giving average contributions of 64.63% and 15.40%, respectively. The exception was that the disposal of the Essix retainer contributes most significantly in the freshwater ecotoxicity category at 57.59%.

### Disability-adjusted life years

The results of DALYs were converted into seconds and are shown in Table 7. The Hawley retainers had the higher DALYs impact equivalent to 4.94 seconds compared with 4.73 seconds for the Essix retainer. Global warming, water consumption, and photochemical ozone formation were the most significant categories contributing to the DALYs impact for both retainers.

**Table 7.**
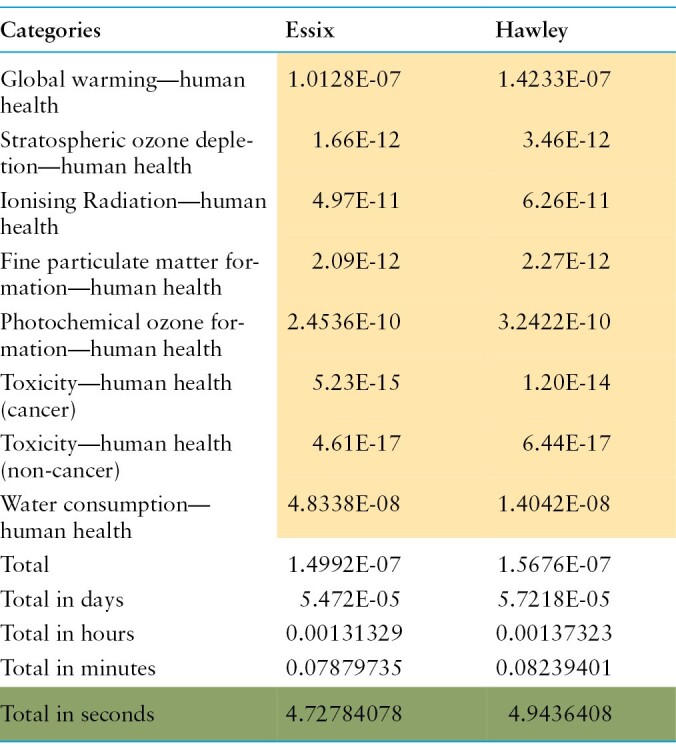
DALYs.

## Discussion

The aim of this study was to determine the environmental sustainability of post-orthodontic retention using dental retainers. In this study, the comparative LCA methodology was utilized to quantify and analyse the environmental impact of the Hawley versus the Essix retainer. As this is a comparative LCA, the environmental impact values are based on the difference values rather than the absolute values.

The Hawley retainer has a greater overall environmental impact as compared to the Essix retainer. In the 16 impact categories, Hawley was worse off than Essix in 12 categories. This is because the Hawley retainer has more components, which would require a greater amount of manufacturing, production, packaging, transportation, and disposal. Substituting the Hawley with Essix retainer would reduce climate change total by 0.4 times and ozone layer depletion by 1.1 times. To put it into context, the disability-adjusted life seconds for Hawley and Essix are 4.94 and 4.73 seconds, respectively. This means that the fabrication of a Hawley and an Essix would result in a loss of life of 4.94 and 4.73 seconds, respectively.

The factory manufacturing and in-house production of the Hawley retainer contributes the lion’s share of the environmental burden. Manufacturing and production account for an average of 41.45% and 52.52% across all impact categories, respectively. Both exceeded their respective averages in 8 of 16 categories. For the Essix retainer, production is the biggest contributor to the environmental burden averaging 64.53% across all impact categories. This percentage was exceeded in 9 of 16 categories.

The environmental burden generated by the Hawley and Essix retainers is mainly in their manufacturing and production stages. One main reason for this is the source of fuel used to generate the energy required. Manufacturing and production occurred in Ireland, other parts of Europe and the UK. The Sustainable Energy Authority of Ireland’s (SEAI) 2020 report states that petroleum oil accounts for 1.7% of Ireland’s energy, while solid fossil fuels like peat and coal account for 9.7% and 3.4%, respectively [22]. Whereas in Europe as of 2019, solid fossil fuel and petroleum oil amount to 12.7% and 36.3%, respectively [23]. As of 2021, the UK’s energy source like coal accounts for only 1% [24]. The combustion of fossil fuels is responsible for greenhouse gas emissions, acid rain, smog, and respiratory illnesses [25]. As a result, this has led to the manufacturing and production processes dominating majority of the impact categories for both retainers.

Currently, there is a plan in place that all Organisation for Economic Co-operation and Development (OECD) nations are to phase out fossil fuels. One example would be for OECD nations to phase out coal use by 2030 [26]. Ireland has kept its promise by closing two peat-fired power plants in 2020 [27]. By phasing out fossil fuels and moving to renewable sources of energy, it would greatly reduce the environmental impacts of production for both the Hawley and Essix retainers. However, it must be noted that the environmental burden caused by manufacturing and production may be overstated and not reflective of the current situation. This is because the Ecoinvent database used for this research is based on data collected in the past. This would not be so accurate for our research in terms of the absolute values as most countries have now moved on from solid fossil fuels to more sustainable sources of energy. Nevertheless, this would not render the findings invalid. This is because, for both retainers, the absolute values would be reduced by an equal proportion in all aspects of the life cycle. As such, the relative difference would remain the same.

There are no similar studies that compare the environmental impacts and sustainability of Hawley versus Essix retainers. However, there is a study that investigated the environmental sustainability of clear aligners. Clear aligners are thermoformed plastic appliances like Essix retainers but have active components. The study highlighted that the disposal of thermoformed plastics causes harm to the environment as the breakdown of plastics can take up to 1000 years and incineration will release toxic fumes [28]. However, the study did not carry out an LCA of clear aligners; therefore, it did not provide any quantifiable data on the environmental burden.

A limitation of this study was that several assumptions were made. For example, some raw materials used to fabricate the retainers were not available in the Ecoinvent database. Hence, suitable alternatives of comparable properties were selected. All transport routes were also assumed to be via the shortest land, sea, and air routes. These assumptions were based on information obtained online. These may result in a slight discrepancy between our assumptions and the actual processes involved in the life cycle of each type of retainer. However, these assumptions are reasonable and supported by research. Therefore, the differences are likely to be minuscule and may not lead to significant changes to the result of this study.

Another limitation of this study was that it is a comparative study. As such, common products, processes, and machines used in the fabrication of both retainers were excluded. Therefore, if a new type of orthodontic retainer was developed, this LCA may not be extrapolated to obtain an environmental impact comparison. This is because the environmental impact results are data of Essix relative to Hawley retainers and do not provide the absolute values from ‘cradle to grave’.

The clinical efficacy, cost-effectiveness, and patient preference should be considered alongside the environmental impacts of the retainers. Both the Hawley and the Essix retainers serve the same purpose of long-term post-orthodontic retention. Systematic reviews by Littlewood *et al.* [29] and Mai *et al.* [30] showed that both retainers possess similar retention characteristics, but some studies state that Essix provides better stability in the lower arch [31]. However, there is no scientific reasoning for Essix providing better lower arch stability. Essix is also found to be better at preventing relapse of incisor irregularity [32]. On the basis of clinical efficacy and environmental sustainability, Essix retainers seem to be the post-orthodontic retainer of choice. But in cases that require retention of rapid maxillary expansion and better occlusal settling, the Hawley retainer needs to be used [33].

In terms of patient satisfaction, the Essix is more aesthetically pleasing and does not interfere with speech. Several studies also reported that patients found Hawley retainers more difficult to wear than Essix retainers. Therefore, patients with Essix retainers are more compliant than patients with Hawley retainers [31].

In terms of cost-effectiveness, a study by the National Health Service (NHS) showed that the cost to the NHS was €152.42 for the Hawley while that for Essix was €121.08. The mean cost to the patient for the Hawley was €9.15 and the Essix was €6.93. Therefore, Essix is more cost-effective for both clinician and patient [34].

An important aspect to investigate is the lifespan of each type of retainer. As post-orthodontic retention is lifelong, retainers need to be replaced if worn, broken, or misplaced. There are limited studies investigating the survival rate of removable retainers [29]. Of these studies, there were no consistent results when comparing the Hawley and Essix retainers. A randomized controlled trial (RCT) by Hichens *et al*. [34] found that the Essix retainer had a longer survival rate than the Hawley retainer over a period of 6 months. However, this may be due to the method used to account for broken retainers. Repeated breakage of one retainer was accounted for as separate failures. This would result in an overestimation in the results. In addition, this study had a short follow-up period of 6 months.

On the other hand, an RCT by Sun *et al*. [35] highlighted that there was no significant difference in survival rates between the two retainers over a 12-month follow-up. However, this study also accounted for the survival time of the 75th percentile for both retainer types. For the Hawley, the maxillary and mandibular had survival times of 344 and 140 days, respectively. For the Essix, the maxillary and mandibular had survival times of 175 and 83 days, respectively. This shows that although the survival rates between the two retainer types at 12 months had no significant difference, Hawley had a longer survival time when compared with Essix within the same time period. Moreover, a 12-month follow-up may not truly reflect the potential lifespan of each retainer type.

A retrospective cohort study by Jin *et al.* [36] found that the Hawley lasted for an average of 1529 days while an Essix lasted around 105 days. For the Hawley, this study only included maxillary retainers. Also, as the data were obtained retrospectively, there may be a potential for bias. However, current prospective studies like the gold standard RCTs have short follow-up periods. In addition, unlike other studies which only provided survival rates, this study provided an absolute value for the survival time of each retainer type. Although Sun *et al.* provided an absolute value, it was for the 75th percentile and only had a 12-month follow-up period. Therefore, the lifespan for both retainers reported by Jin *et al.* was chosen for this comparative LCA.

According to the study by Jin *et al.* [36], the Hawley would last approximately 14.56 times longer than the Essix. As a result, the Essix would be worse off than the Hawley in all 16 impact categories. (Table 8) To put it into context, the Hawley would have a DALYs of 4.94 seconds when compared with the Essix with a DALYs of 68.85 seconds over the same period. Therefore, when taking into account the lifespan, the Hawley retainer would be more environmentally sustainable than an Essix retainer.

**Table 8.** Life-cycle impact assessment results; summary of environmental burdens for the use of Essix or Hawley retainer when factoring in lifespan.

*Impact category*	*Units*	*Essix*	*Hawley*
Non-carcinogenic effects	CTUh	1.01E-07	9.69E-09
Minerals and metals	kg Sb-eq	6.15E-06	7.79E-07
Freshwater ecotoxicity	CTU	1.62962276	0.08261
Climate change total	kg CO_2_-eq	1.58928629	0.15337
Freshwater eutrophication	kg P-eq	3.41E-04	2.25E-05
Respiratory effects—inorganic	Disease incidence	4.83E-08	3.61E-09
Photochemical ozone creation	kg NMVOC—eq	0.00407733	0.00037
Terrestrial eutrophication	mol N-eq	0.01179514	0.00105
Marine eutrophication	kg N-eq	1.29E-03	0.00011
Ozone layer depletion	kg CFC-11-eq	4.56E-08	6.51E-09
Ionising radiation	kg U235-eq	0.08518714	0.00736
Land use	Points	15.6073039	0.83699
Freshwater terrestrial acidification	mol H+-eq	0.00553352	0.00066
Fossils	MJ	34.1093689	2.44299
Carcinogenic effects	CTUh	2.29E-08	3.60E-09
Dissipated water	m^3^ water-eq	0.95744524	0.0191

## Conclusion

In summary, based on the evidence gathered from this comparative LCA, Hawley retainers are more environmentally sustainable than Essix retainers. Furthermore, it is acknowledged that the lifespan of the retainers is a crucial factor in the long term. These results would better enable clinicians to factor in the environmental impact and make informed decisions on the choice of retainer type.

## Supplementary Material

cjae012_suppl_Supplementary_table_1

cjae012_suppl_Supplementary_table_2

cjae012_suppl_Supplementary_table_3

cjae012_suppl_Supplementary_Material

## Data Availability

The data underlying this article are available within the article or its supplementary materials.
