# Supplementary material for: Environmental sustainability of post-orthodontic dental retainers: a comparative life-cycle assessment of Hawley and Essix retainers
Source: Eur J Orthod. 2024 Mar 15;46(2):cjae012. doi: 10.1093/ejo/cjae012 (PMC10941639; doi:10.1093/ejo/cjae012)
Supplement: cjae012_suppl_Supplementary_table_1 [file cjae012_suppl_supplementary_table_1.docx]

*Supplementary Table 1: Assumption and exclusion list*

| Dentist-patient Interaction | - Dental staff and patient travel is excluded from this analysis. This is because it is assumed that patient travel and the staff travel for both retainers are the same as the same number of appointments are needed for both. - Energy and water waste produced during the fitting of each retainer was excluded. - Assume that on fitting appointment, if adjustment required, both retainers are adjusted by using the same acrylic bur. Also, we assumed that in case of an adjustment, both retainers take the same time to be adjusted. Furthermore, we assumed that both retainers are handed to the patient in a plastic box. |
| --- | --- |
| Common Steps in Fabrication | - The common products, processes and machines used in the fabrication of both retainers are excluded. - The common initial steps to manufacture for both retainers assumed to be the same. For both retainers, impressions taken using a tray painted with adhesive and filled with alginate. The impressions are disinfected using cidex. The impressions are poured by using type 3 stone. For both retainers, the casts are mounted using a plaster. |
| Raw Materials | - If the composition of each material used was not available by the manufacturer, the composition was estimated based on alternative manufacturers. |
| Polypropylene Sheet | - The raw material (polypropylene granulate) was purchased from the global market. |
| Powder Acrylic  (Acrylic Polymer) | - Raw materials (polymethyl methacrylate, sodium hydroxide, and hydrogen peroxide) were purchased from the global market. - Raw material (benzoyl peroxide) was not found on ecoinvent so reaction formula was used to estimate the weights of its constituents   - Hydrogen peroxide and sodium hydroxide were both found on ecoinvent but benzoyl chloride was not, we therefore used the reaction formula of benzoyl chloride to estimate the weight of its constituents used to form benzoyl chloride.   - Benzotrichloride was not found on EcoInvent so a suitable alternative (Benzal chloride) was used instead.   - Assumed that all above reactions/formation of raw materials were carried out by the manufacturer (Sigma Aldrich) - Assumed that during production, laboratory technicians use precise amounts of powder and liquid acrylic, so no waste accumulates. |
| Liquid Acrylic  (Acrylic Monomer) | - Excluded N-Dimethyl-p-toluidine as it was not available in openLCA (and neither was any other of its synonyms or similar products) and it was <1% of the composition. - Raw materials were assumed to be imported from the UK. - We opted for the powder form of the product as it is available in ecoinvent in kg, the supplier was able to provide them both in liquid or powder form. - It was assumed that Sigma Aldrich is the supplier of the liquid acrylic to Orthocare. - Assumed that during production, laboratory technicians use precise amounts of powder and liquid acrylic, so no waste accumulates. |
| Modelling Wax | - The raw material (petroleum) assumed to be purchased from the EU market. |
| Stainless Steel | - It was assumed that the stainless-steel wire distributor gets material from the largest stainless-steel producer in the world, which is based in Spain. - It was assumed that the wooden spool used for packaging is made up of birch wood. - Density of the wooden spool used for packaging stainless steel was assumed to be 670 kg/m3. |
| Packaging | - Instruction manuals in all packages were excluded. - The packaging used in the transport of raw materials was excluded. - The packaging for transport of products from manufacturer to distributor was assumed to be similar to the packaging used to transport the products from distributor to DDUH, the disposal was only accounted for once. - All products were assumed to be transported in a 450g corrugated cardboard box. The number of products in each box were assumed based on volumetric calculations. It was assumed that this was the unit of purchase by the DDUH. |
| Manufacturing | - Manufacturing processes for the raw materials were obtained through the manufacturers websites and when not available were assumed by researching processes for the fabrication of similar products. - If a process or machinery was not found in the database, the energy used in kWh was estimated. - Maintenance of machinery was excluded. - Waste generated by machinery in manufacturing was excluded. - For the Essix retainer, the excess acrylic trimmed off after the retainer was thermoformed was assumed to be disposed as municipal waste - For the Hawley retainer, excess stainless-steel that was trimmed off and modelling wax waste were assumed as municipal waste. Powdered and liquid acrylic were assumed to be used in precise amounts, so no waste accumulates. - Fumes generated by making the acrylic base were assumed to be removed by the fume hood as municipal waste. |
| Transport | - Transport routes and methods were assumed using Air Miles Calculator®, Google Maps® and Sea Rates® - For land travel, An EU6 lorry was assumed to transport the product from the manufacturer to the distributor. - Light commercial transport was assumed to be the mode of transport from Dublin port to distributors Irish headquarters to the DDUH. - Sea travel was assumed to be by freight from Holyhead port to Dublin port. |
| Home care and Hygiene | - Assumed that both can be cleaned the same way. |
| Disposal | - It was assumed that both retainers are disposed of in general household waste. |
| Hawley Retainer | - LCA was based on the fabrication of a single Hawley Retainer. |
| Essix Retainer | - LCA was based on the fabrication of a single Essix Retainer. |
