# Supplementary material for: Environmental sustainability of post-orthodontic dental retainers: a comparative life-cycle assessment of Hawley and Essix retainers
Source: Eur J Orthod. 2024 Mar 15;46(2):cjae012. doi: 10.1093/ejo/cjae012 (PMC10941639; doi:10.1093/ejo/cjae012)
Supplement: cjae012_suppl_Supplementary_table_2 [file cjae012_suppl_supplementary_table_2.docx]

*Supplementary Table 2: Life cycle inventory for one hawley retainer*

| **Description** | **Input** | **Output** | **Unit** | | **Provider** |
| --- | --- | --- | --- | --- | --- |
| **Hawley** | | | | | |
| Electricity (medium voltage) | 0.190122 | NA | kWh | | market for electricity, medium voltage \| electricity, medium voltage \| Cutoff, U – IE |
| **Liquid acrylic** | | | | | |
| Ethylene glycol | 0.0352 | NA | g | | market for ethylene glycol \| ethylene glycol \| Cutoff, U – GLO |
| Methacrylic acid | 0.0352 | 0.00081 | g | | market for methacrylic acid \| methacrylic acid \| Cutoff, U – GLO |
| Packaging (aluminium alloy, AlLi) | 0.153 | NA | g | | aluminium alloy production, AlLi \| aluminium alloy, AlLi \| Cutoff, U - RoW |
| Packaging (corrugated cardboard box) | 0.0000132 | NA | Kg | | market for corrugated board box \| corrugated board box \| Cutoff, U – RER |
| Packaging (injection moulding) | 0.153 | NA | g | | market for injection moulding \| injection moulding \| Cutoff, U – GLO |
| Packaging (polypropylene, granulate) | 0.00438 | NA | g | | market for polypropylene, granulate \| polypropylene, granulate \| Cutoff, U – GLO |
| Transport (transport, freight, light commercial vehicle) | .00032358*23 | NA | kg*km | | market for transport, freight, light commercial vehicle \| transport, freight, light commercial vehicle \| Cutoff, U - Europe without Switzerland |
| Transport (transport, freight, lorry 16-32 metric ton, EURO6) | .00032358*25.8 | NA | Kg*km | | market for transport, freight, lorry 16-32 metric ton, EURO6 \| transport, freight, lorry 16-32 metric ton, EURO6 \| Cutoff, U - RER |
| Transport (transport, freight, lorry 16-32 metric ton, EURO6 | .00032358*257 | NA | kg*km | | market for transport, freight, lorry 16-32 metric ton, EURO6 \| transport, freight, lorry 16-32 metric ton, EURO6 \| Cutoff, U - RER |
| Transport (transport, freight, lorry 16-32 metric ton, EURO6) | .00032358*418 | NA | Kg*km | | market for transport, freight, lorry 16-32 metric ton, EURO6 \| transport, freight, lorry 16-32 metric ton, EURO6 \| Cutoff, U - RER |
| Transport (transport, freight, sea, ferry) | .00032358*110 | NA | Kg*km | | market for transport, freight, sea, ferry \| transport, freight, sea, ferry \| Cutoff, U - GLO |
| Municipal solid waste (liquid acrylic) | NA | 1.75919 | g | | market for municipal solid waste \| municipal solid waste \| Cutoff, U - IE |
| Waste packaging (municipal solid waste) | NA | 0.15738 | g | | market for municipal solid waste \| municipal solid waste \| Cutoff, U - IE |
| Waste packaging (paperboard) | NA | 0.0000132 | kg | | market for waste paperboard \| waste paperboard \| Cutoff, U - IE |
| **Beauty wax** | | | | | |
| Petroleum slack wax | 0.6 | NA | g | petroleum slack wax production, petroleum refinery operation \| petroleum slack wax \| Cutoff, U - Europe without Switzerland | |
| Packaging (corrugated cardboard box) | 0.000006 | NA | Kg | market for corrugated board box \| corrugated board box \| Cutoff, U – RER | |
| Packaging (corrugated cardboard box) | 0.000006 | NA | Kg | market for corrugated board box \| corrugated board box \| Cutoff, U – RER | |
| Packaging (sulfate pulp, bleached) | 0.00061 | NA | Kg | market for sulfate pulp, bleached \| sulfate pulp, bleached \| Cutoff, U – RER | |
| Transport (transport, freight, light commercial vehicle) | 0.000622*278 | NA | Kg*km | market for transport, freight, light commercial vehicle \| transport, freight, light commercial vehicle \| Cutoff, U - Europe without Switzerland | |
| Transport (transport, freight, lorry 16-32 metric ton, EURO6) | 6.22E-4*396 | NA | Kg*km | market for transport, freight, lorry 16-32 metric ton, EURO6 \| transport, freight, lorry 16-32 metric ton, EURO6 \| Cutoff, U - RER | |
| Transport (transport, freight, lorry 16-32 metric ton, EURO6) | 0.000622*296 | NA | Kg*km | market for transport, freight, lorry 16-32 metric ton, EURO6 \| transport, freight, lorry 16-32 metric ton, EURO6 \| Cutoff, U – RER | |
| Transport (transport, freight, lorry 16-32 metric ton, EURO6) | 0.000622*135 | NA | Kg*km | market for transport, freight, lorry 16-32 metric ton, EURO6 \| transport, freight, lorry 16-32 metric ton, EURO6 \| Cutoff, U – RoW | |
| Transport (transport, freight, sea, ferry) | 0.000622*110 | NA | Kg*km | market for transport, freight, sea, ferry \| transport, freight, sea, ferry \| Cutoff, U – GLO | |
| Municipal solid waste (beauty wax) | NA | 0.6 | g | market for municipal solid waste \| municipal solid waste \| Cutoff, U - IE | |
| Waste packaging (paperboard) | NA | 0.000622 | g | market for waste paperboard \| waste paperboard \| Cutoff, U - IE | |
| **Powder acrylic** | | | | | |
| Benzyl chloride | 0.133767658 | NA | g | market for benzyl chloride \| benzyl chloride \| Cutoff, U – RER | |
| Hydrogen peroxide, without water, in 50% solution state | 0.056498141 | NA | g | market for hydrogen peroxide, without water, in 50% solution state \| hydrogen peroxide, without water, in 50% solution state \| Cutoff, U – RoW | |
| Neutralising agent, sodium hydroxide-equivalent | 0.0332342 | NA | g | sodium hydroxide to generic market for neutralising agent \| neutralising agent, sodium hydroxide-equivalent \| Cutoff, U – GLO | |
| Polymethyl methacrylate, beads | 4.2465 | NA | g | market for polymethyl methacrylate, beads \| polymethyl methacrylate, beads \| Cutoff, U – GLO | |
| Packaging (corrugated cardboard box) | 0.000018625 | NA | Kg | market for corrugated board box \| corrugated board box \| Cutoff, U - RER | |
| Packaging (polyethylene terephthalate, granulate, bottle grade) | 123.15/9000*4.47/1000 | NA | Kg | market for polyethylene terephthalate, granulate, bottle grade \| polyethylene terephthalate, granulate, bottle grade \| Cutoff, U – GLO | |
| Transport (transport, freight, light commercial vehicle) | 7.97895E-5*23 | NA | Kg*km | market for transport, freight, light commercial vehicle \| transport, freight, light commercial vehicle \| Cutoff, U - Europe without Switzerland | |
| Transport (transport, freight, lorry 16-32 metric ton, EURO6) | 7.97895E-5*418 | NA | Kg*km | market for transport, freight, lorry 16-32 metric ton, EURO6 \| transport, freight, lorry 16-32 metric ton, EURO6 \| Cutoff, U – RER | |
| Transport (transport, freight, lorry 16-32 metric ton, EURO6) | 7.97895E-5*25.8 | NA | Kg*km | market for transport, freight, lorry 16-32 metric ton, EURO6 \| transport, freight, lorry 16-32 metric ton, EURO6 \| Cutoff, U – RER | |
| Transport (transport, freight, lorry 16-32 metric ton, EURO6) | 7.97895E-5*257 | NA | Kg*km | market for transport, freight, lorry 16-32 metric ton, EURO6 \| transport, freight, lorry 16-32 metric ton, EURO6 \| Cutoff, U – RER | |
| Transport (transport, freight, sea, ferry) | 7.97895E-5*110 | NA | Kg*km | market for transport, freight, sea, ferry \| transport, freight, sea, ferry \| Cutoff, U – GLO | |
| Municipal solid waste (powder acrylic) | NA | 4.47 | g | market for municipal solid waste \| municipal solid waste \| Cutoff, U - IE | |
| Waste packaging (paperboard) | NA | 0.000018625 | Kg | market for waste paperboard \| waste paperboard \| Cutoff, U - IE | |
| Waste packaging (polyethylene) | NA | 123.15/9000*4.47/1000 | kg | market for waste polyethylene \| waste polyethylene \| Cutoff, U - IE | |
| **Stainless steel** | | | | | |
| Hot rolling, steel | 0.36 | NA | g | hot rolling, steel \| hot rolling, steel \| Cutoff, U - Europe without Austria | |
| Sheet rolling, chromium steel | 0.36 | NA | g | market for sheet rolling, chromium steel \| sheet rolling, chromium steel \| Cutoff, U - GLO | |
| Steel, chromium steel 18/8 | 0.36 | NA | g | market for steel, chromium steel 18/8 \| steel, chromium steel 18/8 \| Cutoff, U - GLO | |
| Wire drawing, steel | 0.36 | NA | g | market for wire drawing, steel \| wire drawing, steel \| Cutoff, U – GLO | |
| Packaging (corrugated cardboard box) | 0.000004 | NA | Kg | market for corrugated board box \| corrugated board box \| Cutoff, U - RER | |
| Packaging (packaging film, low density polyethylene) | 11.26/450*0.32/1000 | NA | Kg | market for packaging film, low density polyethylene \| packaging film, low density polyethylene \| Cutoff, U – GLO | |
| Packaging (pulpwood, hardwood, measured as solid wood under bark) | 0.1976/670000 | NA | m^3^ | market for pulpwood, hardwood, measured as solid wood under bark \| pulpwood, hardwood, measured as solid wood under bark \| Cutoff, U - Europe without Switzerland | |
| Transport (transport, freight, light commercial vehicle) | .0000797895*25.8 | NA | Kg*km | market for transport, freight, light commercial vehicle \| transport, freight, light commercial vehicle \| Cutoff, U - Europe without Switzerland | |
| Transport (transport, freight, light commercial vehicle) | .0000797895*23 | NA | Kg*km | market for transport, freight, light commercial vehicle \| transport, freight, light commercial vehicle \| Cutoff, U - Europe without Switzerland | |
| Transport (transport, freight, lorry >32 metric ton, EURO6) | .0000797895*197 | NA | Kg*km | market for transport, freight, lorry >32 metric ton, EURO6 \| transport, freight, lorry >32 metric ton, EURO6 \| Cutoff, U – RER | |
| Transport (transport, freight, lorry >32 metric ton, EURO6) | .0000797895*257 | NA | Kg*km | market for transport, freight, lorry >32 metric ton, EURO6 \| transport, freight, lorry >32 metric ton, EURO6 \| Cutoff, U – RER | |
| Transport (transport, freight, sea, container ship) | .0000797895*278 | NA | Kg*km | market for transport, freight, sea, container ship \| transport, freight, sea, container ship \| Cutoff, U – GLO | |
| Municipal solid waste (stainless steel) | NA | 0.36 | g | market for municipal solid waste \| municipal solid waste \| Cutoff, U - IE | |
| Waste packaging (paperboard) | NA | 0.000004 | kg | market for waste paperboard \| waste paperboard \| Cutoff, U - IE | |
| Waste packaging (polyethylene) | NA | 11.26/450*0.32/1000 | kg | market for waste polyethylene \| waste polyethylene \| Cutoff, U - IE | |
| Waste packaging (wood, untreated) | NA | 0.1976 | g | market for waste wood, untreated \| waste wood, untreated \| Cutoff, U - IE | |
