# Supplementary material for: Environmental sustainability of post-orthodontic dental retainers: a comparative life-cycle assessment of Hawley and Essix retainers
Source: Eur J Orthod. 2024 Mar 15;46(2):cjae012. doi: 10.1093/ejo/cjae012 (PMC10941639; doi:10.1093/ejo/cjae012)
Supplement: cjae012_suppl_Supplementary_table_3 [file cjae012_suppl_supplementary_table_3.docx]

*Supplementary Table 3: Life cycle inventory of an essix retainer*

| Description | Input | Output | Unit | Provider |
| --- | --- | --- | --- | --- |
| **Essix** | | | | |
| Electricity, medium voltage | 0.00754 | NA | kWh | market for electricity, medium voltage \| electricity, medium voltage \| Cutoff, U - IE |
| **Acrylic sheet** | | | | |
| Polypropylene, granulate | 20.26 | NA | g | market for polypropylene, granulate \| polypropylene, granulate \| Cutoff, U – GLO |
| Thermoforming, with calendering | 20.26 | NA | g | market for thermoforming, with calendering \| thermoforming, with calendering \| Cutoff, U - GLO |
| Packaging (corrugated cardboard box) | 0.0001342 | NA | Kg | market for corrugated board box \| corrugated board box \| Cutoff, U – RER |
| Packaging (corrugated cardboard box) | 0.00375 | NA | Kg | market for corrugated board box \| corrugated board box \| Cutoff, U – RER |
| Packaging (packaging film, low density polyethylene) | 0.0005008 | NA | Kg | market for packaging film, low density polyethylene \| packaging film, low density polyethylene \| Cutoff, U – GLO |
| Packaging (packaging film, low density polyethylene) | 0.00153 | NA | kg | market for packaging film, low density polyethylene \| packaging film, low density polyethylene \| Cutoff, U – GLO |
| Transport (transport, freight, light commercial vehicle) | .00591*11 | NA | Kg*km | market for transport, freight, light commercial vehicle \| transport, freight, light commercial vehicle \| Cutoff, U - Europe without Switzerland |
| Transport (transport, freight, lorry 16-32 metric ton, EURO6) | 0.00591*22 | NA | Kg*km | market for transport, freight, lorry 16-32 metric ton, EURO6 \| transport, freight, lorry 16-32 metric ton, EURO6 \| Cutoff, U - RER |
| Transport (transport, freight, lorry 16-32 metric ton, EURO6) | .00591*290 | NA | Kg*km | market for transport, freight, lorry 16-32 metric ton, EURO6 \| transport, freight, lorry 16-32 metric ton, EURO6 \| Cutoff, U - RER |
| Transport (transport, freight, lorry 16-32 metric ton, EURO6) | .00591*461 | NA | Kg*km | market for transport, freight, lorry 16-32 metric ton, EURO6 \| transport, freight, lorry 16-32 metric ton, EURO6 \| Cutoff, U – RoW |
| Transport (transport, freight, sea, ferry) | .00591*110 | NA | Kg*km | market for transport, freight, sea, ferry \| transport, freight, sea, ferry \| Cutoff, U - GLO |
| Municipal solid waste (acrylic sheet) | NA | 20.26 | g | market for municipal solid waste \| municipal solid waste \| Cutoff, U - IE |
| Waste packaging (paperboard) | NA | 0.00388 | Kg | market for waste paperboard \| waste paperboard \| Cutoff, U - IE |
| Waste packaging (polyethylene) | NA | 0.00203 | kg | market for waste polyethylene \| waste polyethylene \| Cutoff, U - IE |
