# Supplementary material for: Environmental sustainability of post-orthodontic dental retainers: a comparative life-cycle assessment of Hawley and Essix retainers
Source: Eur J Orthod. 2024 Mar 15;46(2):cjae012. doi: 10.1093/ejo/cjae012 (PMC10941639; doi:10.1093/ejo/cjae012)
Supplement: cjae012_suppl_Supplementary_Material [file cjae012_suppl_supplementary_material.docx]

*Supplementary List 4: Abbreviations*

LCA: Life Cycle Assessment

HSE: Health Service Executive

DALYs: Disability-adjusted life years

DDUH: Dublin Dental University Hospital

kWh: Kilowatt-hour

ISO: International Organization for standardisation

PEF: Product Environmental Footprint

LCIA: Life Cycle Impact Analysis

OECD: Organisation for Economic Co-operation and Development

NHS: National Health Service
